# Mass Antibiotic Treatment Alone Does Not Eliminate Ocular Chlamydial Infection

**DOI:** 10.1371/journal.pntd.0000394

**Published:** 2009-03-31

**Authors:** Paul M. Emerson, Jeremiah Ngondi

**Affiliations:** 1 The Carter Center, Atlanta, Georgia, United States of America; 2 Department of Public Health and Primary Care, Institute of Public Health, University of Cambridge, Cambridge, United Kingdom; Weill Medical College of Cornell University, United States of America

Trachoma is caused by *Chlamydia trachomatis* and is the leading infectious cause of preventable blindness globally. For trachoma control, the World Health Organization (WHO) promotes the SAFE strategy, an integrated program of 1) eyelid **S**urgery to correct the in-turned eyelashes associated with severe trachoma and stops pain and minimizes risk of corneal damage; 2) **A**ntibiotic treatment for active trachoma using single-dose oral azithromycin or tetracycline eye ointment; 3) **F**acial cleanliness through sustained behavior change to reduce transmission; and 4) **E**nvironmental improvement, to increase access to water and sanitation [1],[2]. The A, F, and E components of SAFE have been designed to treat ocular Chlamydia infection and reduce the risk of trachoma transmission, such that blinding trachoma is eliminated.

There has been considerable debate as to whether mass treatment with antibiotics alone can eliminate trachoma. In Tanzania, a study by Solomon et al., conducted in a single isolated village, showed that one mass treatment with azithromycin and periodic tetracycline eye ointment had reduced ocular infection with *C. trachomatis* from 9.5% to 0.1% 24 months post-treatment [3]. Following a second round of mass azithromycin treatment at 24 months, no *C. trachomatis* DNA was detected on ocular swabs from the entire community at 60 months after baseline [4]. In The Gambia, a study of 12 villages reported that a single mass treatment with azithromycin was probably sufficient for long-lasting control of *C. trachomatis*; however, re-infection occurred through contact with untreated communities [5]. In contrast, a second single village study from Tanzania showed that after two rounds of mass treatment with azithromycin, ocular infection with *C. trachomatis* was still present, and neither clinical trachoma nor infection had been eliminated [6],[7].

The paper by Lakew et al. provides the best data yet contributing to the elimination debate [8]. It is an excellent 42-month longitudinal assessment of ocular Chlamydia infection following four biannual mass treatments, at months 0–18 in 16 communities in Gurage zone of Southern Nations Nationalities and Peoples Region, a trachoma hyper-endemic area of Ethiopia. The authors have previously reported findings from studies conducted in the same study area: four concluded that local elimination of ocular Chlamydia infection in hyper-endemic areas was feasible with biannual mass treatment with azithromycin [9]–[12]; one found that infection was not eliminated after a single dose of mass azithromycin [13]; and two indicated that mass treatment with azithromycin provided protection for both treated and untreated individuals [14],[15].

Unlike previous studies in Tanzania and The Gambia, the Lakew et al. [8] study includes more villages (16), conducted biannual mass treatment with azithromycin, and provided a follow-up of 42 months. There was considerable variation between the villages and none of them performed exactly as the mean of the 16. In other words, there was no “average” or “typical” village. This argues strongly that findings from the single village studies [4],[6],[7] should be interpreted with caution, or even considered as anecdotal.

The field teams did an excellent job in achieving and documenting high coverage with antibiotics, and attained impressive results. The mean study coverage was over 90% with 100% of the eligible population reported for several villages. Although the current WHO guidelines suggest aiming for “a minimum” of 80% coverage, programs should not consider this as the goal, but aim for 100% and document the true coverage. It may be possible for programs to routinely achieve above 85% coverage—the Lakew et al. study suggests that this would greatly improve impact.

This study used four rounds of antibiotic distributed 6 months apart. Decline in infection was rapid and deep (from an average of 63.5% to 2.6%). Yet, the rebound was also steep, roughly doubling at each successive 6-month period after treatment stopped to 25.2% after 24 months. During the course of the study nine of 16 villages achieved local elimination of ocular Chlamydia in children (i.e., a prevalence of infection of 0%). Importantly, none of these villages maintained a 0% prevalence through to the 42-month follow-up, demonstrating that local elimination is not the same as sustainable control as previously suggested [9]–[12]. Re-infection took place from somewhere, and once it did, transmission was rapid, as the behaviors and environmental conditions that favored trachoma had not been altered. *The message for trachoma programs is very clear: however much we may be tempted to, if we base trachoma control in hyper-endemic areas solely on antibiotic distribution, success is not likely*.

Operational decisions by national trachoma control programs are made on the basis of clinical signs of trachoma and not nucleic acid amplification tests (NAATs). The merits of NAATs compared to clinical signs remains a controversial issue. A recent review by Wright and Taylor critically explored the discrepancies that exist between the clinical signs and NAATs and attributes this largely to the kinetics of the disease [16]. Although laboratory tests have a role in complementing clinical signs in monitoring antibiotic treatment, they have limitations because of their exorbitant costs and impracticality under field conditions. Yet, all national trachoma control programs have access to clinical signs that require only a visual inspection of the tarsal conjunctiva, while none routinely uses NAATs. Lakew et al. provided prevalence of clinical signs for the baseline survey, but not for the follow-up surveys. Inclusion of both trachomatous inflammation-follicular (TF) and trachomatous inflammation-intense (TI) as separate indicators for the follow-up results may have helped elucidate our understanding of the disparity between these signs and positivity by NAAT. Additionally, it would have been interesting to see the effect of the multiple treatments on the clinical signs, and on TI in particular since it appears most susceptible to intervention [17],[18]; is associated with higher infection rates [19] and a greater load of *C. trachomatis* DNA [20],[21]; and is more closely linked to progression to cicatricial trachoma [22].

There is an idea that three annual rounds of azithromycin will result in elimination of ocular infection and districts can “graduate” from implementation programs. Forecasts for global azithromycin demand are based on this assumption. These data do not support that idea. Programs should not expect to graduate districts after 3 years, but should reassess the trachoma prevalence after at least 3 years and expect to continue implementation rather than stop at the risk of losing the ground gained. The Pfizer donation of azithromycin is greatly appreciated and has been tremendously important in breathing life into trachoma control programs. As the country programs expand to recruit more people and aim for elimination of blinding trachoma, the global demand for donated azithromycin is increasing. The 135 million doses pledged in 2003 have been consumed, and there has been no clear public declaration for a continued and expanded donation—although the annual quantity of azithromycin generously provided by Pfizer has continued to rise in 2009. Should the capacity of programs to use azithromycin exceed the capacity of the donor to produce it, there will be a need for decisions on the most rational use of the resource to be made. Options for the most rational use of azithromycin may include focusing on the most endemic countries; restricting treatment to children (the reservoir of infection); an attack phase of annual treatment for 3 years followed by a maintenance phase of treatment once in every 2 years; or basing decisions to treat on TI rather than TF. The data provided by Lakew et al. show that arbitrary graduation after three annual treatments alone is not one of them.

There is little doubt that the mass distribution of azithromycin for trachoma control is the most effective way of rapidly reducing ocular infection with *C. trachomatis*, and that mass distribution will probably have many population level collateral benefits beyond trachoma control. However, unless accompanied by effective facial cleanliness and environmental improvements, mass treatment alone will not result in eliminating trachoma in the most affected areas. The vast data provided by the team in Gurage zone are a sobering indication that we cannot rely on drug distribution alone to eliminate transmission of trachoma and that there remains an urgent need for substantial investment in hygiene promotion, water, and sanitation in addition to drug distribution to release the world from the tragedy of blinding trachoma.
